# Clinical characteristics, management, and outcomes of sepsis in Lusaka, Zambia

**DOI:** 10.1186/cc10409

**Published:** 2011-10-27

**Authors:** SM Chimese, B Andrews, S Lakhi

**Affiliations:** 1 Department of Internal Medicine, University of Zambia, Lusaka, Zambia; 2 Institute for Global Health, Vanderbilt University, Nashville, TN, USA; 3 Department of Internal Medicine, University Teaching Hospital, Lusaka, Zambia

## Introduction

Although infectious diseases are the leading causes of death in sub-Saharan Africa, there are few studies describing sepsis in the region. Available data suggest that HIV prevalence is disproportionately high among septic patients and that treatment, particularly fluid administration, may be suboptimal [1]. Our study evaluated the clinical characteristics, management, and hospital outcomes of patients admitted with sepsis in Zambia. We hypothesized that patients with bacteremia have higher in-hospital mortality than those without.

## Methods

We conducted a prospective observational study of patients admitted with sepsis to the Adult Filter Clinic (medical ER) of the University Teaching Hospital (UTH) in Lusaka Zambia. Sepsis was defined as two or more SIRS criteria and clinically suspected infection. Baseline characteristics and laboratory results were recorded, as was the timing of antibiotics and fluid administration. Patients were followed until discharge or death.

## Results

In 3 months, 161 septic patients were enrolled. One hundred and ten (68%) patients were HIV positive; 23 (14%) had unknown HIV status. Ninety-one (57%) had severe sepsis. Organ dysfunction included altered mentation (31%), renal dysfunction (16%), severe respiratory distress (respiratory rate ≥40) (11%), thrombocytopenia (11%), and hepatic dysfunction (7%). Multiple organ dysfunction occurred in 26%. After excluding contaminants, blood cultures were positive in 29 (18%) patients. *Staphyloccoccus aureus*, salmonella species, *Streptococcus pneumoniae*, and *Klebsiella pneumoniae *were the most common pathogens. Only 29% of patients received intravenous fluids within 1 hour of presentation. Eighty-four percent of patients received ≤1 l within the first 6 hours of presentation, and 55% received ≤1 l in the first 24 hours. Overall in-hospital mortality was 40.4% (65/161). In-hospital mortality for severe sepsis was 54.9% (50/91). Important predictors for in-patient mortality (Table 1) were low Glasgow Coma Scale on admission (adjusted odds ratio (AOR) 16.0 (2.9 to 87.1)), positive blood culture (AOR 4.8 (1.5 to 15.0)), and positive and unknown HIV status (AOR 4.20 (1.0 to 17.0) and AOR 7.7 (1.2 to 47.7), respectively).

**Table 1:** Risk factors for in-hospital death

Variable	Adjusted OR	Crude OR
Anemia, Hb <9 g/dl	0.91 (0.74 to 4.93)	1.05 (0.56 to 1.80)
Blood culture positive	**4.8 (1.50 to 15.0)**	**2.38 (1.14 to 4.95)**
GCS		
≥13	1	1
9 to 12	**5.50 (1.90 to 16.20)**	**3.60 (1.60 to 8.11)**
<9	**16.00 (2.90 to 87.10)**	**11.2 (3.50 to 36.4)**
HIV		
Negative	1	1
Positive	**4.20 (1.00 to 17.00)**	**2.35 (0.88 to 6.28)**
Unknown	**7.70 (1.20 to 47.70)**	**8.38 (2.36 to 29.7)**
<1 hour to IVF	0.40 (0.10 to 1.10)	0.86 (0.43 to 1.72)
MAP <65	2.10 (0.70 to 6.80)	1.25 (0.56 to 2.81)
IVF in first 6 hours		
0	1	1
1	0.80 (0.30 to 2.00)	0.81 (0.41 to 1.61)
2 or more	0.30 (0.10 to 1.10)	0.60 0.23 to 1.58)

Statistically significant ORs in bold.

## Conclusion

In-hospital mortality due to sepsis is higher in Zambia than in most studies from the developed world. Low Glasgow Coma Scale and positive blood cultures are associated with increased in-hospital mortality. Insufficient i.v. fluid administration probably contributes to the high overall mortality. Standardized management including early fluids and antibiotics might improve outcomes of sepsis and severe sepsis in sub-Saharan Africa.
